# The Relation of the So-Called Minor Gynecological Operations to Intrapelvic Inflammation

**Published:** 1892-09

**Authors:** L. S. McMurtry

**Affiliations:** Louisville, Ky., Fellow of the American Association of Obstetricians and Gynecologists; 231 West Chestnut Street


Buffalo Medical and Surgical Journal

Vol. XXXII. SEPTEMBER, 1892. No. 2.

### Original Communications.

### THE RELATION OF THE SO-CALLED MINOR GYNECOLOGICAL OPERATIONS TO INTRAPELVIC INFLAMMATION.

By L. S. McMURTRY, M. D., of Louisville, Ky.,

Fellow of the American Association of Obstetricians and Gynecologists.

The diagnosis of intrapelvic diseases, their treatment during the primary or formative stages, and decision of the vital question of surgical interference, constitute a large and important share of the responsible duties of the practitioner of medicine. The advance of American civilization, with the growth of large cities and departure from the simple habits of agricultural life, with an accumulating influx of European pauper population and an increase of vice, have diffused and augmented the prevalence of specific inflammatory diseases of the pelvic organs. The ready infection of the intraperitoneal structures during an unprotected labor or puerperium is so common, that no one can fail to recognize this as a conspicuous cause of intrapelvic inflammatory disease.

There is another factor in the etiology of intrapelvic inflammation which is not so generally recognized, and which is of the highest practical importance. In the light of the advances made in pelvic surgery, it has become evident that a large proportion of the cases of intrapelvic inflammation ending in structural alterations requiring surgical treatment, are the direct results of certain manipulations and modes of treatment in common use to relieve comparatively unimportant affections of the pelvic organs. In this way it often obtains that the physician, in his efforts to relieve some comparatively trivial disorder, inflicts upon the patient a more serious malady. Such treatment, in the majority of instances, is based upon certain fallacies as to the pathology of pelvic diseases, some of which it will be my endeavor in this paper to point out. Surely no subject can deserve, both for improvement in our thera-

1. Read before the Medical Society of the County of Erie, at Buffalo, N.Y., June 14, 1892.

peutic resources and for our own conscientious enlightenment, more careful consideration.

### THE PERNICIOUS EFFECTS OF UNNECESSARY EXAMINATIONS.

It is an error to assume that every young woman who complains of pain in the back and headache at the menstrual period is the subject of uterine or ovarian disease. These phenomena are part of the constitutional disturbance incident to this important function, and are more or less violent in accordance with the temperament and habits of the individual, and the systemic condition prevailing at the time. A saline cathartic and warm sitz baths to relieve the pressure of congestion, together with rest in bed, will generally beget speedy relief and put the patient on her feet in comfort. How common it is for such cases to be treated with repeated and increasing doses of opium we all know, thus impeding the secretions, arresting elimination, interfering with digestion, and protracting an indisposition which, with ordinary care, will correct itself in a short time.

But of more serious concern is the assumption that the cause of dysmenorrhea consists in a mechanical impediment to the menstrual flow. I do not wish to be understood as implying that mechanical dysmenorrhea does not occur. On the contrary, obstruction and retention from imperforate hymen and cervical stenosis do obtain, but the proportion of these cases is very limited, and the symptoms so marked and persistent that their significance cannot be overlooked. The routine practice of resorting to an elaborate examination, with speculum and sound, in these cases is unjustifiable. The custom of making examinations of young girls, with the speculum or otherwise, by mere fact of menstrual pain or irregularity, is more than an error; it is a grievous wrong which cannot be condoned or excused. I would not consume time upon this point were it not that cases are so frequently brought to my knowledge wherein these unnecessary examinations and manipulations are resorted to, producing distressing mental depression and humiliation, as well as pernicious physical effects. This practice, so harmful in its results, and based upon principles quite erroneous, is not common with specialists in gynecology, but is more frequently resorted to by amateur gynecologists engaged also in general practice. When it is clearly necessary to explore the pelvic organs in a young unmarried woman, she should be anesthetized for that purpose.

That the uterine sound is not essential for accurate diagnosis; that it may be the bearer of infection to the receptive endometrium; that it often denudes the mucous membrane, and may readily penetrate the uterine walls and pass into the peritoneal cavity, are facts now generally recognized, and which were first brought specifically to the attention of the profession by Dr. William Warren Potter, the distinguished president of this society.

THE USE OF CAUSTICS AND CHEMICAL SOLUTIONS.

After the numerous papers and discussions in special and general medical societies, widely published, during the past two years, upon the evil effects of caustic agents applied to the mucous surface of the cervix and body of the uterus, one would suppose this practice would be abandoned by progressive physicians. Yet such is not the case. This mode of treatment is in general favor in treating various forms of pelvic disease, and is erroneously called conservative treatment. I could recite here case after case, from my own practice, showing beyond question that mild forms of pelvic inflammation have been converted into serious and acute grades by this treatment, and that disease of the tubes and ovaries dated its activity from the time such treatment was instituted.

Emmet, long ago, pointed out the evils of intrauterine medication, but his teachings have not been heeded by the great body of the profession, though confirmed by many careful observers. It is a common practice, constantly fraught with danger, to apply strong caustic and astringent agents to the endometrium,—such as iodine, nitrate of silver, carbolic acid, etc.,—with the idea of producing some indefinite alterative effect upon the pelvic organs, and thereby relieve certain obscure symptoms.

The vaginal douche is in general use, and, in appropriate application, is of inestimable value in the treatment of pelvic congestion and recent inflammation. It is too frequently advised without regard to the menstrual period (when it should never be used), and without detailed and specific advice as to the manner of its application. When used with too great force, with an old or foul syringe, and with strong chemical solutions, it may readily infect lymphatic channels leading to the peritoneum. The treatment of simple leucorrhea as a substantive disease, with strong chemical solutions, in this way, can readily establish tubal disease and associated pelvic peritonitis. By applying caustic agents to the endometrium, traumatic inflammation is established in the immediate vicinity of peritoneal channels.

### DILATATION OF THE CERVIX UTERI.

Forcible dilatation of the cervix is accomplished in a variety of ways, but, in the majority of cases, is unnecessary by any method. This operation is resorted to for dysmenorrhea and for sterility, upon an assumed diagnosis of stenosis of the cervical canal as the cause of one or both of these conditions. I have already endeavored to show in this paper, that the diagnosis in the majority of cases is only an assumption, and not a deduction from positive indications. This operation is performed by sponge and tupelo tents, by graduated hard-rubber bougies, and by powerful steel instruments. In these days of cleanliness in surgery, it would be natural to presume that no one would venture to use upon a surface in close proximity to, and in direct vascular communication with, the peritoneum such an offensive thing as a sponge tent. Yet these tents are favorite instruments for dilating the cervical canal with some practitioners. They are prepared with an odor of carbolic acid, and thereby give a deceptive sense of security and confidence.

Within twenty-four hours after the introduction of these tents the patient's pulse is quickened, the temperature is elevated, and she complains of pain in the back and pelvic region. She suffers an attack of pelvic peritonitis, which gradually subsides in its acute symptoms, but exhibits its sequelæ later in a displaced and fixed uterus, with chronic inflammation of the uterine appendages. This is a familiar observation of gynecologists, and I could cite numerous cases that have come under my own care with advanced tubo-ovarian disease directly traceable to the use of sponge tents. The danger from repeated use of the graduated hard-rubber dilators is a traumatism, which may readily extend itself through communicating channels to the peritoneum.

But the favorite instrument for dilatation is the steel dilator, with its powerful blades, usually used during anesthesia, which wounds extensively the mucous surface of the cervical canal and tears asunder the muscular fibers of the cervix. Wonders have been claimed for this instrument. It has been recommended for many varieties of inflammatory disease of the uterus, and it has been claimed that dilatation by this instrument will cure menstrual disorders and nervous disturbances associated therewith. This operation has been likened unto the operation for divulsion of the sphincter ani muscle for the cure of ulceration of the rectal mucous membrane and associated reflexes. There is no analogy between the organs or the conditions involved. I have known women of highly-endowed nervous sensibilities, who, from the imperfect development of the uterus and adnexa, resulting from idiosyncrasy and the defective hygiene of boarding-school life, suffer severely at the menstrual periods, treated by forcible dilatation under a mistaken idea that the small cervical canal was the cause of all trouble. Of course, no good could come from such an erroneous conception of the troubles incident to an undeveloped uterus and its adnexa, and much harm results from the traumatism. That dilatation of the cervical canal, by a gentle hand, protected by thorough aseptic methods, may serve a useful purpose as a preparatory step to the removal of pathological growths within the uterine cavity, is apparent ; but for nervous symptoms and dysmenorrhea, and in the treatment of sterility, this operation has no place, and its persistent use is a common and prolific cause of disease of the uterine appendages.

### INDISCRIMINATE TRACHELORRAPHY.

One of the greatest advances of modern gynecology was made by Emmet when he demonstrated that the condition so long regarded and treated as ulceration of the cervix uteri was in reality a laceration of that structure which had deviated from the physiological course of repair after labor. The operation known as trachelorraphy, or Emmet's operation, is, in appropriate application, one of the most efficient and conservative known to gynecic surgery ; and yet I know no operation which misapplied has done, and is now doing, so much mischief. It is, indeed, a great improvement in our pathology and practice to recognize these lesions as traumatic, and restore the integrity of the structures involved, instead of tormenting the patient and increasing the inflammatory area by the use of cautery and powerful astringents. Moreover, the cervix is relieved by excision of the cicatricial deposit, so prone to malignancy, and of the contraction so painful in its reflexes.

This operation restores the patient to health, and cures sterility, which is so frequently associated with the deeply-torn and ulcerating cervix. But the operation has been more widely abused than any other operation in gynecology. The rents made during labor by the passing head, which mark every multiparous cervix, and which are within the physiological limit, have been pared and sewed as a remedy for every variety of pelvic disease and almost every form of nervous disorder. The results have, naturally, been disappointing. But, what is worse, these operations have, in a large proportion of cases, been done in appropriate cases, so far as the lesions themselves were concerned, but where the cervical lesions were complicated by unrecognized intrapelvic inflammation, and where careless operative methods have started an inflammatory process involving the appendages, thus causing a more serious condition of disease than the operation was undertaken to cure. In many instances, by this operation, a physiological laceration, unindurated and healed, is laid open, and suppuration follows with failure of union and an open ulcer ; in other cases, a subacute inflammation in the uterine appendages is rekindled into an active process, ending in fixation and, possibly, suppuration.

These results of unskilful and inappropriate application of this very useful operation are very common, and I venture to assert that not a single practitioner here, who sees much of pelvic disease, can but bear testimony to the fact from his own weekly experience. For my own part, the connection between the operation and the lesions of tubes and ovaries is so common that the narrative of symptoms by the patient is almost uniform and characteristic. Thorough asepsis during operations in and near the cervix, and protection from infection afterward, require skilled attention and precautions not readily secured.

### THE ROUTINE USE OF THE CURETTE.

One of the so-called minor operations most popular nowadays is the operation of curettement. For the removal of fungous growths from the endometrium it has long been in use. Its efficiency in the treatment of these growths, as well as in removing detritus of all kinds from the interior of the uterus, is well established. But its application has been greatly extended by recent teachings as to the pathology of endometritis. That the endometrium should require, when inflamed, such aggressive treatment as will result in its destruction, I am unable to understand. The indiscriminate use of the curette, followed by local application of powerful chemicals, such as tincture of iodine and pure carbolic acid, for simple catarrhal inflammation, has no scientific basis, and that it is often the cause of severe grades of inflammation of the tubes and ovaries, I have seen abundant clinical evidence to satisfy any one. Simple endometritis is a rare disease, and is usually associated with active inflammatory changes in the appendages and adjacent peritoneum. To treat the endometrium by curettement and caustics amid such complications, will surely aggravate the intrapelvic disease. In the early stage of endometritis, before the inflammatory process has extended to the appendages, all that treatment can accomplish may be had by thorough cleansing, and this should be done with the utmost care.

It was my purpose to call attention to the pernicious results of electropuncture and aspiration in the treatment of pelvic diseases, but these methods, so frequently condemned, are now becoming generally recognized as dangerous, and scarcely require specific mention.

In conclusion, I beg to submit the following summary of the points presented in this thesis :

1. The etiology of intrapelvic inflammation (salpingitis, ovaritis, and peritonitis,) may be three-fold : (1) puerperal, (2) specific, (3) post-operative (traumatic).

2. Unnecessary and uncleanly examinations, with introduction of the sound, may cause, by traumatism and infection, pelvic inflammation.

3. Forcible dilatation, with steel instruments, sponge tents, and other instruments, may beget intrapelvic inflammatory disease. This operation has a very narrow sphere of utility, and is usually performed upon erroneous pathological data.

4. Operations on the cervix, with associated disease of the appendages, is dangerous. The treatment of lacerations by caustics and astringents is never satisfactory and always dangerous. Trachelorraphy is an operation of high utility, but requires discrimination in application and skill in execution in order to obtain good results. It is often the initial step in tubo-ovarian disease of severe type.

5. Curettement, while of unquestioned value in removing neoplasms and detritus from the endometrium, is abused as a method of treating inflammatory conditions of the pelvic organs. The curette is an instrument capable of causing extensive lesions that may light up an inflammation extending to the appendages and peritoneum, or aggravate a preëxisting inflammation therein.

231 West Chestnut Street.

---

The Cincinnati Hospital Library has been established, containing 7,363 bound volumes, and 151 current journals. This enterprise is to be commended.

